# Phytochemical profile and antioxidant activity of almond (*Prunus amygdalus* Batsch.) cultivars from northwestern Iran

**DOI:** 10.1038/s41598-026-50377-6

**Published:** 2026-04-25

**Authors:** Marzieh Babashpour-Asl

**Affiliations:** Department of Horticultural Science, Mar.C., Islamic Azad University, Maragheh, Iran

**Keywords:** Almond cultivars, Phenolic compounds, Antioxidant activity, HPLC profiling, Nutritional diversity, Biochemistry, Plant sciences

## Abstract

The phytochemical diversity and antioxidant potential of five almond (*Prunus amygdalus* Batsch.) cultivars from northwestern Iran were systematically investigated. To our knowledge, this study provides the first integrated comparison of phenolic compounds, vitamins, antioxidant activity, and individual phenolics quantified by HPLC in these local Iranian cultivars. Significant cultivar-dependent variation was observed across all measured traits. Among the cultivars, “Yamatga Shabestar” exhibited the highest total phenolic content (1.07 mg GAE g⁻¹ DW), along with elevated levels of flavonoids, tannins, and α-tocopherol, and the strongest antioxidant activity measured by the ferric reducing antioxidant power (FRAP) assay. (0.010 mmol Fe²⁺ g⁻¹ DW). In contrast, “Azar Tasuj” showed consistently lower values (0.12 mg GAE g⁻¹ DW and 0.002 mmol Fe²⁺ g⁻¹ DW). HPLC analysis identified seven major phenolic compounds, with gallic acid reaching 13.5 mg 100 g⁻¹ DW and quercetin enriched in “Yamatga Shabestar,” while catechin was most abundant in “Sangi Azarshahr.” Multivariate analysis further distinguished “Yamatga Shabestar” as metabolically distinct and closely associated with phenolic, and antioxidant related traits. However, given the influence of environmental variability and the limited sampling scope, these findings should be interpreted as a preliminary data survey. Overall, the results suggest the strong potential of “Yamatga Shabestar” as a cultivar–environment combination for future breeding-oriented studies and for the development of functional food and nutraceutical applications.

**Supplementary Information:**

The online version contains supplementary material available at 10.1038/s41598-026-50377-6.

## Introduction

 The almond (*Prunus amygdalus* Batsch., syn. *Prunus dulcis* Mill.) is widely regarded as one of the most important nuts due to its nutritional, economic, and health-promoting properties^1^. As a rich source of plant proteins, unsaturated fatty acids, dietary fiber, vitamins, and phenolic compounds, almonds serve a dual role as both a staple food and a functional ingredient in human diets^2^. Globally, almond cultivation has expanded considerably, with major production concentrated in regions such as North America, the Mediterranean basin, and parts of Asia^3^. Within this context, Iran occupies a unique position: it is not only among the leading producers of almonds but also recognized as a primary center of genetic diversity for the species^4,5^. Iran is recognized as one of the important almond-producing countries worldwide. According to FAOSTAT data, national almond production exceeded 130,000 tons in recent years, positioning Iran among the leading global producers^6^. In addition to widely cultivated commercial cultivars such as “Mamayi”, “Rabi”, and “Shahrood”, numerous traditional landraces are maintained in specific agro-ecological zones, particularly in northwestern Iran, where they contribute substantially to national agrobiodiversity and represent valuable genetic resources for breeding and research programs^7^. Beyond their macronutrient composition, almonds are an abundant source of diverse phytochemicals, including phenolic acids, flavonoids, and tannins^8^. These secondary metabolites are of particular interest due to their ability to enhance not only the nutritional quality of almonds but also their medicinal potential. Phenolic compounds, for instance, are concentrated in the almond skin and contribute significantly to its antioxidant capacity^9^. Flavonoids and tannins exert additional biological activities, such as anti-inflammatory, antimicrobial, and cardioprotective effects^10,11^. A growing body of epidemiological evidence suggests that regular almond consumption is associated with reduced incidence of chronic disorders, including cardiovascular disease, type 2 diabetes, and certain cancers, positioning almonds as a functional food of considerable therapeutic relevance^1,12^.

The biological importance of almond phytochemicals is largely attributed to their antioxidant activity and their ability to modulate oxidative stress–related processes. Oxidative stress, resulting from an imbalance between reactive oxygen species (ROS) and endogenous antioxidant defenses, plays a central role in the development of chronic diseases such as atherosclerosis, diabetes, and cancer^13^. Almonds are particularly rich in natural antioxidants, which collectively contribute to free radical scavenging and redox homeostasis. Phenolic compounds, largely concentrated in the almond skin, have been reported as major contributors to antioxidant capacity, while α-tocopherol represents the predominant lipid-soluble antioxidant in the kernel^14–16^. The phytochemical composition and antioxidant capacity of almonds are not uniform traits but are strongly influenced by genetic background and environmental conditions. Substantial cultivar-dependent variation has been reported in total phenolic content, flavonoid profile, tocopherol concentration, and overall antioxidant activity, with values often differing by several-fold among genotypes^17,18^. Such variability highlights the importance of cultivar-oriented studies aimed at identifying nutritionally superior almond genotypes and supports the growing interest in almonds as functional foods with potential benefits for human health and disease prevention.

Environmental conditions such as altitude, temperature, precipitation, and soil properties further modulate the biosynthesis and accumulation of these secondary metabolites^19^. Such variability highlights the importance of evaluating almond germplasm within specific agro-ecological contexts. Regional assessments of local landraces can therefore identify cultivars with superior nutritional and functional attributes, providing valuable resources for breeding programs, genetic conservation, and the development of high-value functional foods.

Although considerable research has been devoted to the phytochemical composition and antioxidant activity of tree nuts such as walnuts, pistachios, and almonds in different countries, comprehensive comparative analyses of local almond cultivars within Iran remain limited. Previous investigations have often focused on commercial varieties, thereby neglecting the rich genetic diversity of traditional landraces that thrive under specific ecological conditions^20,21^. Moreover, few studies have critically examined how environmental variability, such as altitude, climate, or soil mineral composition, interacts with genetic factors to influence almond phytochemistry, leaving important gaps in understanding the mechanisms driving chemical diversity. From a global perspective, comparative studies across regions are essential to contextualize local findings and identify universal versus environment-specific phytochemical trends^22^. Despite the global diversity of almond germplasm, data from Iranian landraces are underrepresented in international databases, limiting our understanding of how local genotypes contribute to worldwide variation. Therefore, incorporating data from Iran, as one of the primary centers of almond diversity, strengthens the global framework of almond phytochemical research while addressing regional gaps. In this context, the present study provides a comparative characterization of five local almond (*Prunus amygdalus* Batsch.) landraces from northwestern Iran by integrating morphological evaluation with comprehensive phytochemical profiling (total phenolics, flavonoids, tannins, carotenoids, B-group vitamins, α-tocopherol, and HPLC-identified phenolics) and antioxidant capacity (FRAP), with the objective of identifying cultivars with superior nutraceutical potential.

## Materials and methods

### Chemicals and reagents

All reagents used in the quantification of phenolic compounds (gallic acid, Folin–Ciocalteu reagent, and sodium carbonate), flavonoids (rutin, aluminum chloride, sodium nitrite, and sodium hydroxide), antioxidant activity (TPTZ reagent and iron (III) chloride), total carotenoids (acetone), and total tannins (diethyl ether) were purchased from Merck. To enable the precise identification of individual compounds via HPLC, high-purity standard samples were obtained from Sigma-Aldrich.

### Plant material

**Fig. 1:**
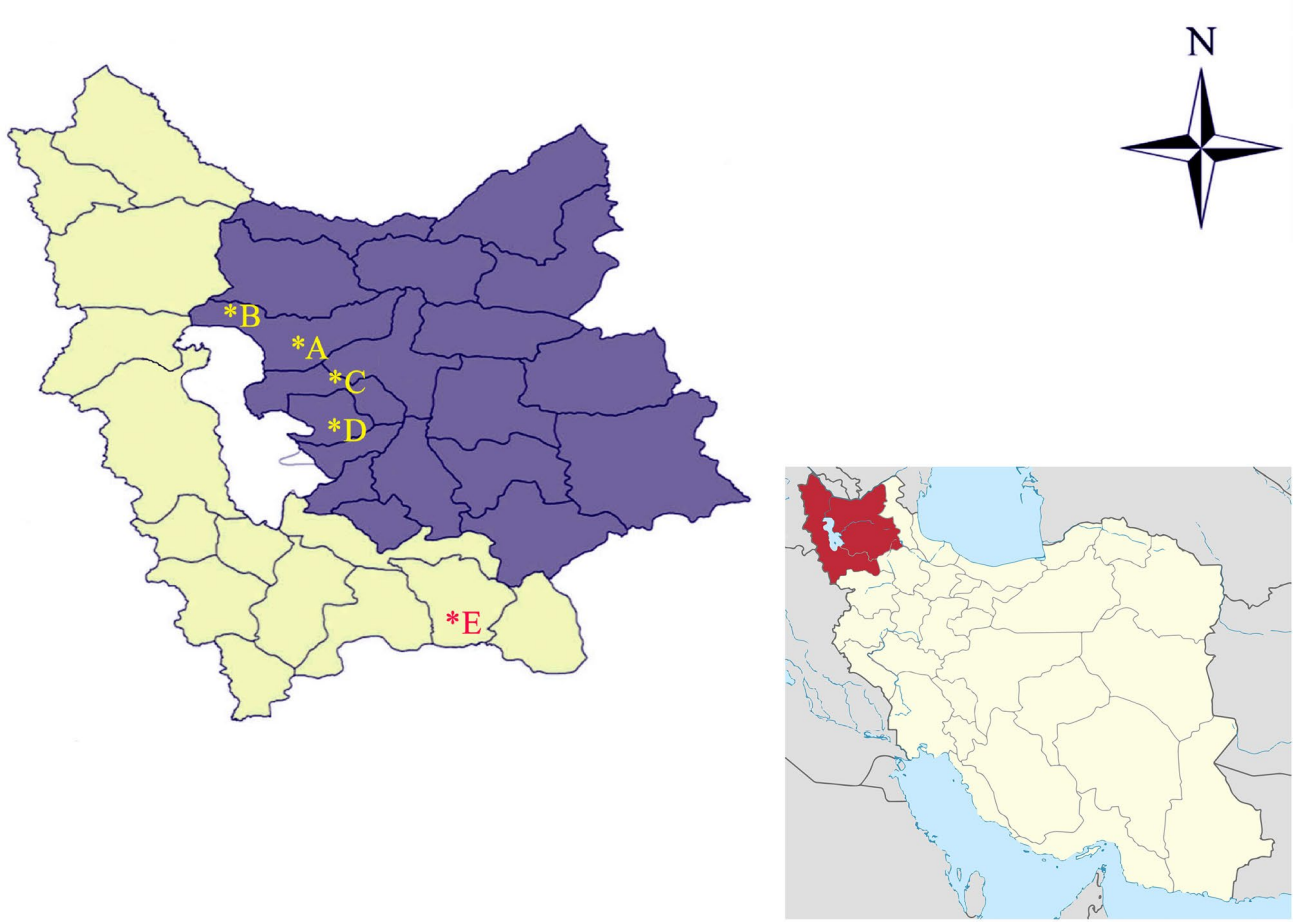
Geographical distribution of the five almond cultivars (*Prunus amygdalus*) sampled from northwestern Iran. Sampling sites are indicated by asterisks: (**A**) Yamatga Shabestar; (**B**) Azar Tasuj; (**C**) Sahand Ilkhchi; (**D**) Sangi Azarshahr; and (**E**) Sangi Shahin Dej. The main map shows West and East Azerbaijan provinces (purple and light yellow), and the inset map shows the location of this region within Iran. Cardinal directions are indicated by the compass rose.

**Fig. 2:**
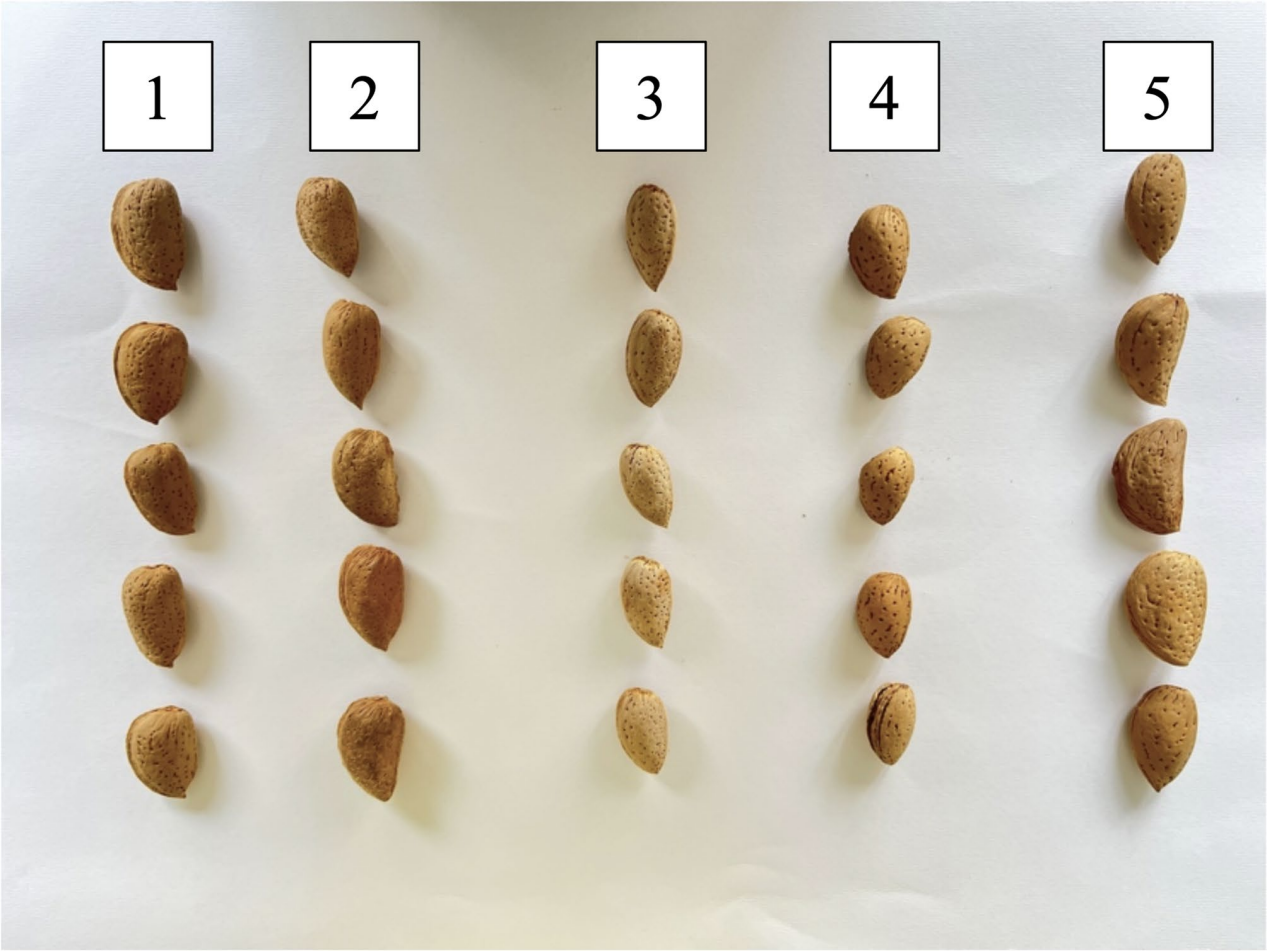
Representative images of the five almond cultivars evaluated in the present study: (1) Sangi Shahin Dej, (2) Sangi Azarshahr, (3) Yamatga Shabestar, (4) Sahand Ilkhchi, and. (5) Azar Tasuj. Whole fruits are shown to illustrate morphological variation among cultivars.

In the present study, almond samples were collected at commercial maturity in September 2025 from five distinct geographical regions, namely Azarshahr, Ilkhchi, Shabestar, Tasuj, and Shahin Dej. The almond cultivars selected for this study represent traditional local landraces that are widely cultivated in specific regions of northwestern Iran and have been maintained through farmer selection over long periods. The cultivar–region associations were as follows: ‘Sangi Azarshahr’ was collected from Azarshahr, ‘Sahand Ilkhchi’ from Ilkhchi, ‘Yamatga Shabestar’ from Shabestar, ‘Azar Tasuj’ from Tasuj, and ‘Sangi Shahin Dej’ from Shahin Dej. These genotypes were chosen due to their local importance, adaptation to regional agro-climatic conditions, and limited representation in previous phytochemical studies. A widely commercialized reference cultivar was not included in the present work, as the primary objective was to characterize and valorize underexplored local germplasm rather than to perform direct comparisons with commercial cultivars. Each almond cultivar was sampled from a single representative cultivation region. Thus, each genotype was associated with a specific geographical location, reflecting the traditional cultivation pattern of these local cultivars in northwestern Iran. The geographical distribution of the sampling sites is illustrated in Fig. 1. The collected almond cultivars were botanically authenticated by Dr. Marzieh Babashpour-Asl, Department of Horticultural Science, Islamic Azad University, Maragheh, Iran. Voucher specimens of each almond cultivar were preserved and archived in the departmental reference collection of the Department of Horticultural Science, Islamic Azad University, Maragheh, Iran, for future reference and traceability. All experimental procedures involving plant materials in this study complied with the relevant national and international guidelines and legislation. Almond samples were collected from cultivated farms in northwestern Iran with the consent of the orchard owners. No endangered or protected species were involved. The collected samples were subsequently transferred to the laboratory for further preparation and extraction procedures. Detailed environmental characteristics of the sampling sites, including altitude, temperature, soil type, pH, and annual rainfall, are summarized in Table 1. Climatic variables (temperature and rainfall) represent 10-year long-term averages (2015–2024) obtained from the Iranian Meteorological Organization, rather than data from the single sampling year. Official finalized climatic datasets for the complete year 2025 were not yet available at the time of analysis; therefore, long-term averages were used to ensure a more stable and representative environmental characterization of each region. In order to minimize potential confounding factors, rigorous efforts were made to ensure that the sampled trees were uniform with respect to age (all trees were 12 years old), irrigation regime, and fertilization practices. General fertilization consisted of annual application of nitrogen (120 kg N/ha as urea), phosphorus (80 kg P₂O₅/ha as triple superphosphate), and potassium (100 kg K₂O/ha as potassium sulfate). Samples were collected only from healthy trees, free from pests and diseases, and the fruits selected for analysis were taken exclusively from these healthy trees (Fig. 2). For each almond cultivar, samples were collected from five independent trees (biological replicates), and five fruits were harvested from each tree. This resulted in a total of 25 fruits per cultivar, which were subsequently processed and analyzed for all biochemical assays. Therefore, the observed variations reflect the combined influence of genotype and local environmental conditions, as genotype and environment effects were not experimentally separated in the present design. The collected samples were first assessed for morphological traits, including fruit and almond kernel length, width, and weight. Almond kernels were freeze-dried until constant weight to determine dry weight (DW). The dried material was then ground into a fine powder and prepared for subsequent extraction and biochemical analyses. For methanolic extraction, 500 mg of the powdered kernel was combined with 10 mL of pure methanol. The mixture was sonicated for 20 min, followed by centrifugation at 4,400 rpm for 15 min. Methanol was selected as the extraction solvent for total phenolic content, total flavonoid content, antioxidant activity (FRAP), and HPLC phenolic profiling, as it is widely recognized as an efficient solvent for the extraction of polar phenolic compounds and provides good comparability with previous studies. The primary focus of the present work was the characterization of phenolic compounds and related antioxidant properties. All analyses were performed on almond kernels with the brown skin (testa) intact, as phenolic compounds are known to be predominantly concentrated in this tissue. It should be noted that carotenoids and vitamin E were extracted using solvent systems appropriate for non-polar compounds (acetone for carotenoids and ethanol/hexane for vitamin E), as described in the corresponding subsections, following established and referenced protocols. The resulting supernatant was carefully collected and stored under refrigeration until further analyses. It should be noted that the use of methanol as the sole extraction solvent may limit the recovery of certain compounds with different polarities. Methanol was selected because it is widely used for the extraction of polar phenolic compounds and allows methodological comparability with previous studies.

**Table 1:** Geographical distribution of sampling sites in northwestern Iran, showing the traditional cultivation district of each almond cultivar. Each genotype was sampled from a single location.

Sampling site	Altitude (m)	Mean temperature (°C)	Soil type	pH	Annual rainfall (mm)
*Azarshahr*	1478	11.9 ± 0.5	Clay loam	7.5	240 ± 12
*Ilkhchi*	1244	13.6 ± 0.3	Loamy clay	7.6	291 ± 23
*Shabestar*	1498	14.7 ± 0.9	Sandy loam	7.7	199 ± 49
*Tasuj*	1369	14.8 ± 0.6	Loam	7.6	147 ± 46
*Shahin Dezh*	1407	12.6 ± 0.7	Loamy sand	7.9	192 ± 37

Values represent mean ± SD of measured environmental parameters. Soil types are classified according to USDA texture classification. Climatic data represent 10-year long-term averages obtained from the Iranian Meteorological Organization.

### Morphological measurements

Fruit weight and kernel weight were measured using a digital analytical balance with a precision of 0.0001 g. Fruit length, fruit width, kernel length, and kernel width were determined using a digital caliper with a precision of 0.01 mm. For each cultivar, measurements were performed on 25 fruits collected from five biological replicates (five fruits per tree). Mean values were calculated for each trait and used for subsequent statistical analysis.

### Total phenol and total flavonoid contents measurement

Total phenolic content (TPC) and total flavonoid content (TFC) were determined according to the methods described by Velioglu et al.^23^ and Zhishen et al.^24^, respectively, with minor modifications. TPC was quantified using the Folin–Ciocalteu assay. Briefly, the extract was mixed with Folin–Ciocalteu reagent followed by sodium carbonate, and the resulting blue chromophore was measured spectrophotometrically at 760 nm. Gallic acid was used as the reference standard, and calibration curves were constructed using five standard concentrations ranging from 10 to 200 mg L⁻¹. Results were expressed as milligrams of gallic acid equivalents per gram of dry weight (mg GAE g⁻¹ DW). The calibration curve had an R² linearity of 0.992. TFC was determined using the aluminum chloride colorimetric method. The extract was reacted with aluminum chloride and associated reagents to form stable complexes, and absorbance was recorded at 510 nm. Rutin was used as the external standard, and calibration curves were prepared using five concentrations ranging from 5 to 100 mg L⁻¹. Results were expressed as milligrams of rutin equivalents per gram of dry weight (mg RE g⁻¹ DW). The calibration curve had an R² linearity of 0.988.

### Total antioxidant activity

The antioxidant potential of the extracts was evaluated using the ferric reducing antioxidant power (FRAP) assay according to Benzie and Strain^25^. Briefly, 200 µL of the extract was mixed with 3 mL of freshly prepared FRAP reagent containing TPTZ, FeCl₃·6 H₂O, and acetate buffer (pH 3.6). The reaction mixture was incubated at 37 °C for 10 min to allow formation of the blue ferrous–TPTZ complex. Absorbance was measured at 593 nm using a spectrophotometer. Quantification was performed using FeSO₄·7 H₂O as the external standard. Calibration curves were constructed using five standard concentrations ranging from 100 to 1000 µM. Antioxidant activity was expressed as millimoles of Fe²⁺ equivalents per gram of dry weight (mmol Fe²⁺ g⁻¹ DW). The calibration curve had an R² linearity of 0.986.

### Total carotenoids

Carotenoids were extracted from powdered almond kernels using acetone as solvent, according to established procedures for carotenoid analysis in foods described by Rodriguez-Amaya^26^, with minor modifications. Specifically, 500 mg of the sample was mixed with 10 mL of acetone and vortexed for 2 min, followed by centrifugation at 4,000 rpm for 15 min. The supernatant was collected, and absorbance was measured at 450 nm. Quantification was performed using β-carotene as the external reference standard. Calibration curves were constructed using five standard concentrations ranging from 5 to 50 mg L⁻¹. Total carotenoid content was expressed as micrograms of β-carotene equivalents per 100 g dry weight (µg β-carotene equivalents 100 g⁻¹ DW). The calibration curve had an R² linearity of 0.989.

### Total tannins

Total tannin content was determined according to the method of Makkar et al.^27^. Briefly, 500 mg of the powdered sample was extracted with 10 mL diethyl ether under continuous shaking for 20 min. The extract was centrifuged at 4,000 rpm for 10 min, and the supernatant analyzed spectrophotometrically at 280 nm. Concentrations were calculated using a tannic acid standard curve, allowing precise quantification of total tannins. Because these spectrophotometric assays rely on different reaction mechanisms and calibration standards, the resulting values represent relative indices rather than strictly comparable absolute phenolic quantities.

### HPLC analysis of phenolic compounds

Phenolic compounds were quantified by HPLC using a Knauer system (Germany) equipped with a Waters 2695 separation module, a C18 column (250 × 4.6 mm, 5 μm), and a Waters 2487 UV detector. The selected phenolic compounds were chosen based on previous reports identifying them as the predominant and most frequently detected phenolics in almond kernels and skins. These compounds represent key subclasses of almond phenolics, including phenolic acids (gallic acid, caffeic acid, p-coumaric acid), flavanols (catechin), flavonols (quercetin and kaempferol), and flavonoid glycosides (rutin), and are commonly used as marker compounds in almond phytochemical profiling studies^28^. The mobile phase consisted of methanol (A) and distilled water (B), both containing 0.02% trifluoroacetic acid, with a flow rate of 0.5 mL min⁻¹. The gradient program was set as follows: 20% A/80% B at 0 min, 30:70 at 10 min, 50:50 at 20 min, maintained for 20 min, 100% A within 2 min and held for 6 min, followed by re-equilibration to the initial conditions within 7 min. The injection volume was 20 µL, and the column temperature was maintained at 25 °C. Detection was performed in the range of 200–600 nm. Identification of individual phenolics was achieved by comparison of retention times with authentic standards obtained from Sigma-Aldrich (Buchs, Switzerland). Quantification of gallic acid, catechin, caffeic acid, rutin, kaempferol, p-coumaric acid, and quercetin was performed using external calibration with the corresponding analytical standards (≥ 98% purity) obtained from Sigma-Aldrich. Calibration curves were initially prepared over a broad concentration range; however, quantitative analysis was performed within the linear working range of each compound (5–250 mg L⁻¹), where optimal detector response and linearity were achieved. Quantification was carried out using external calibration curves constructed from authentic standards at seven concentration levels (5–250 mg L⁻¹). All calibration curves showed good linearity, with coefficients of determination (R²) ranging from 0.984 to 0.999 (Table S1). Phenolic concentrations were expressed as mg 100 g⁻¹ dry weight (DW).

### Determination of B-group vitamins

B-group vitamins (thiamine, riboflavin, pantothenic acid, and pyridoxine) were quantified following AOAC^29^ with modifications after Akintimehin et al.^30^. Briefly, thiamine (B1) was extracted in 0.1 N HCl, hydrolyzed at 100 °C, and clarified by centrifugation and filtration after treatment with ethanol, potassium ferricyanide, and toluene. Riboflavin (B2) was extracted in acetic acid solution (50:50, v/v) under similar hydrolysis conditions, followed by filtration. Pyridoxine (B6) was hydrolyzed in 0.1 N HCl, enzymatically digested with takadiastase, and precipitated with trichloroacetic acid prior to clarification. Absorbance values for spectrophotometric quantification were recorded at 530 nm (B1), 461 nm (B2), and 290 nm (B6) using a UV–Vis spectrophotometer. Quantification was based on calibration curves prepared from authentic standards (Sigma-Aldrich), and results were expressed as µg/100 g DW. Pantothenic acid (vitamin B5) was quantified by HPLC under chromatographic conditions optimized for water-soluble vitamins. Separation was performed on a reversed-phase C18 column (250 × 4.6 mm, 5 μm) maintained at 30 °C, using an isocratic mobile phase consisting of 20 mM potassium dihydrogen phosphate buffer (pH 3.0, adjusted with phosphoric acid) and methanol (95:5, v/v) at a flow rate of 1.0 mL min⁻¹. The injection volume was 20 µL. Detection was carried out at 205 nm. Identification was achieved by comparing retention time with an authentic pantothenic acid calcium salt standard (≥ 98%, Sigma-Aldrich, St. Louis, MO, USA), and quantification was performed using external calibration over the concentration range of 0.5–50 mg L⁻¹ (R² ≥ 0.984). Results were expressed as µg 100 g⁻¹ DW.

### Determination of Vitamin E (α-tocopherol)

Vitamin E (α-tocopherol) content was determined using a spectrophotometric method based on the protocol described by Kamal-Eldin and Appelqvist^31^, with minor modifications. Almond kernels were freeze-dried to constant weight and ground into a fine powder prior to extraction. To minimize oxidation and light-induced degradation, all procedures were performed under reduced light conditions. Briefly, 1 g of the powdered sample was extracted with 10 mL of ethanol: hexane (1:1, v/v) under continuous shaking for 1 h at room temperature. The mixture was centrifuged at 4,000 rpm for 10 min, and the upper hexane layer containing α-tocopherol was carefully collected. Absorbance was measured at 292 nm using a UV–Vis spectrophotometer. Quantification was performed using external calibration with authentic α-tocopherol standards (Sigma-Aldrich), and results were expressed as mg 100 g⁻¹ dry weight (DW).

### Statistical analysis

The collected data were subjected to analysis of variance (ANOVA) using SAS software. Mean comparisons were carried out using Duncan’s multiple range test (DMRT) at the 5% significance level (*p* ≤ 0.05). Correlation heatmap matrices were generated in R software, while biplot analysis was performed using OriginPro. To avoid ambiguity in data presentation, the units of measurement were reported according to the analytical approach applied. Extract-based assays, including total phenolic content, total flavonoids, total tannins and antioxidant activity (FRAP), were expressed on a dry weight basis as mg g⁻¹ DW (or mmol g⁻¹ DW for FRAP). In contrast, nutritionally relevant compounds commonly reported in food composition databases, such as vitamins, total carotenoids and HPLC-quantified phenolics, were expressed as mg or µg per 100 g DW. This unit selection reflects standard reporting practices for each analytical category.

## Results

### Morphological traits

**Fig. 3:**
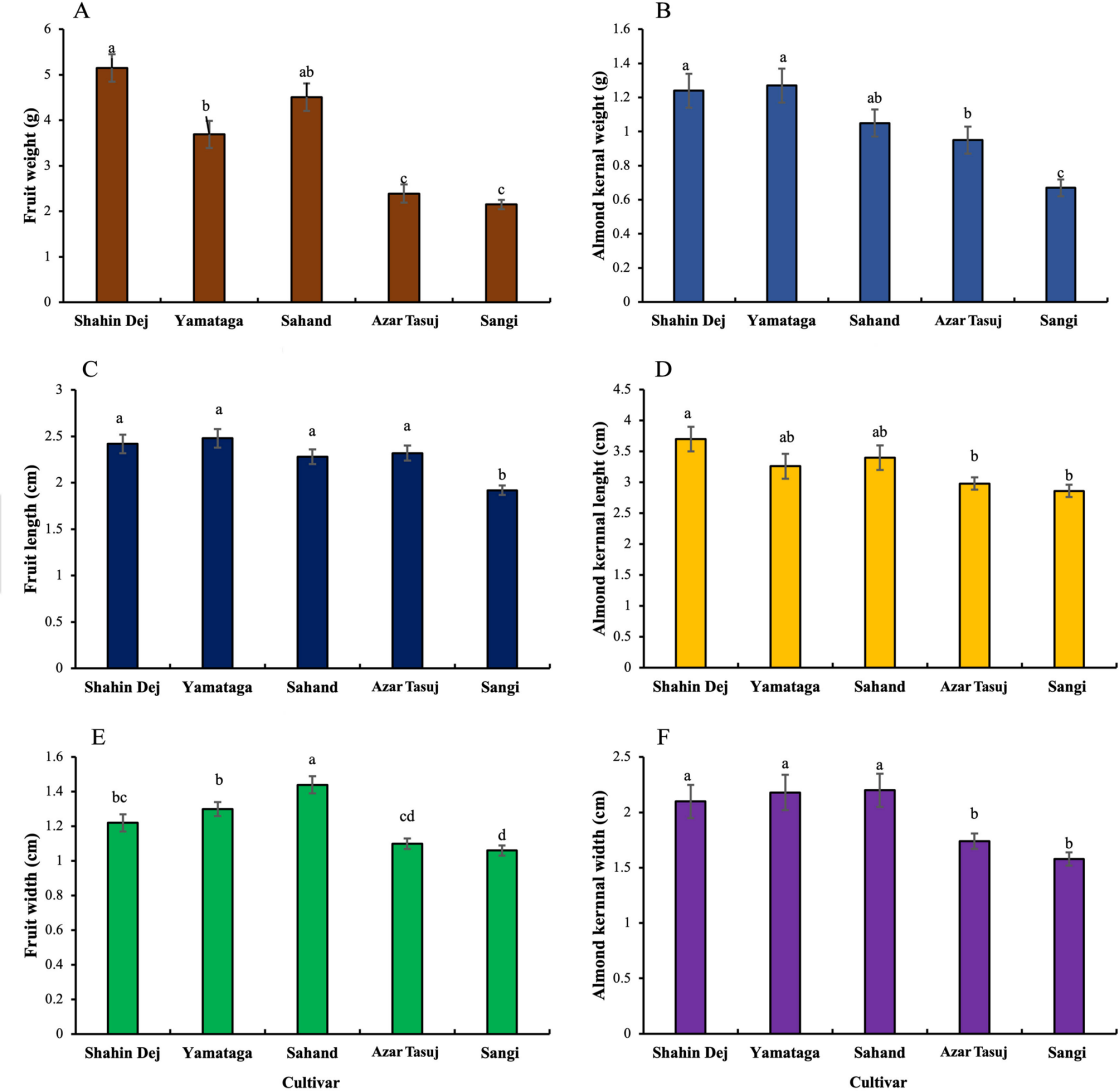
Physical characteristics of five Iranian almond cultivars. Six key traits of fruits and kernels are shown: (**A**) Fruit weight (g), (**B**) Almond kernel weight (g), (**C**) Fruit length (cm), (**D**) Almond kernel length (cm), (**E**) Fruit width (cm), and (**F**) Almond kernel width (cm). Bars represent mean ± SD, (*n* = 25), and different lowercase letters (a–e) indicate significant differences among cultivars (*p* < 0.05, Duncan’s multiple range test).

Cultivar type exerted a significant influence on all measured morphological traits. Fruit weight (F = 9.76, *p* < 0.01), kernel weight (F = 7.25, *p* < 0.01), fruit width (F = 13.75, *p* < 0.01), and kernel width (F = 19.50, *p* < 0.01) differed significantly, while fruit length (F = 3.29, *p* < 0.05) and kernel length (F = 3.73, *p* < 0.05) were also significant. The extensive variation observed among the five cultivars is notable. The heaviest fruits were observed in “Sangi Shahin Dej” (5.15 g), whereas “Sangi Azarshahr” produced the lightest (2.13 g). Kernel weight varied markedly, ranging from 0.67 g in “Sangi Azarshahr” to 1.27 g in “Yamatga Shabestar”. Fruit length was greatest in “Yamatga Shabestar” (2.48 cm) but only 1.92 cm in “Sangi Azarshahr”. Kernel length followed a similar trend, with “Sangi Shahin Dej” producing the longest kernels (3.70 cm), contrasting with “Sangi Azarshahr” (2.86 cm). In terms of fruit and kernel width, “Sahand Ilkhchi” ranked highest (1.44 and 2.20 cm, respectively), whereas “Azar Tasuj” had the narrowest fruits (1.09 cm) and kernels (1.74 cm) (Fig. 3).

### Total phenolic content (TPC)

**Fig. 4:**
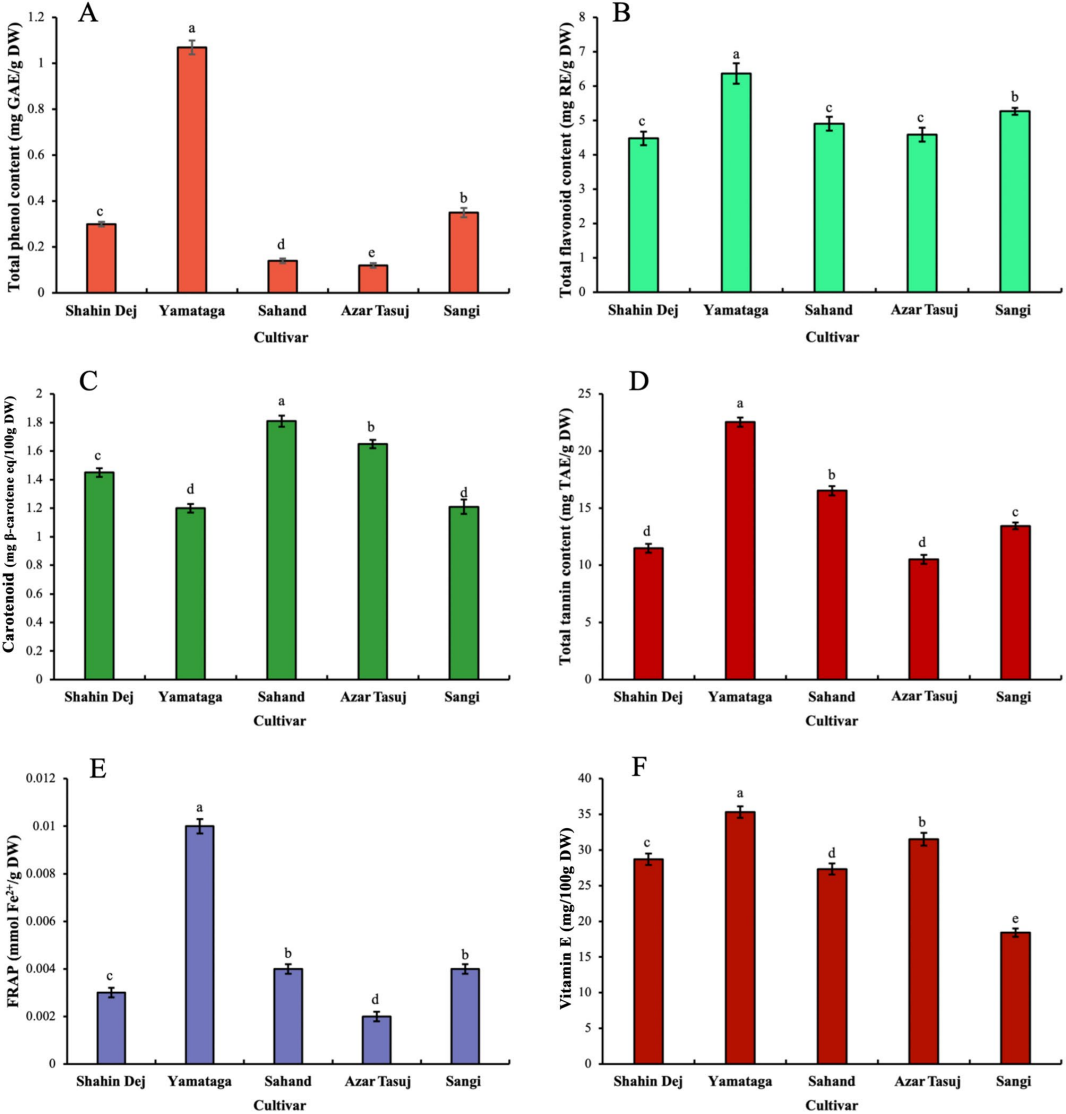
Phytochemical and antioxidant properties of five Iranian almond cultivars. (**A**) Total phenolic content (mg GAE/g DW), (**B**) Total flavonoid content (mg RE/g DW), (**C**) Total carotenoid content (µg β-carotene eq/100 g DW), (**D**) Total tannin content (mg TAE/g DW), (**E**) Ferric Reducing Antioxidant Power (FRAP, mmol Fe²⁺/g DW), and (**F**) Vitamin E content (mg/100 g DW) are shown. Error bars represent mean ± SD (*n* = 25), and different lowercase letters (a–e) indicate statistically significant differences among cultivars (*p* < 0.05, Duncan’s multiple range test).

Unless otherwise stated, extract-based phytochemical and antioxidant parameters are reported as mg g⁻¹ dry weight (DW). Significant differences were also evident in phenolic content (F = 2250.00, *p* < 0.01). Post-hoc analysis (e.g., Duncan’s test) clearly distinguished “Yamatga Shabestar” with the highest TPC (1.07 mg GAE g⁻¹ DW). This represents a difference of approximately 8-fold compared to the lowest cultivar. Intermediate concentrations were recorded in “Sangi Shahin Dej” (0.30 mg GAE g⁻¹ DW) and “Sangi Azarshahr” (0.35 mg GAE g⁻¹ DW). The lowest levels were found in “Sahand Ilkhchi” (0.14 mg GAE g⁻¹ DW) and “Azar Tasuj” (0.12 mg GAE g⁻¹ DW), as illustrated in Fig. 4-A.

### Total flavonoid content (TFC)

Flavonoid content varied significantly across cultivars (F = 72.33, *p* < 0.01). The highest TFC was measured in “Yamatga shabestar” (6.37 mg RE g⁻¹ DW). The lowest values were observed in “Sangi Shahin Dej” (4.48 mg RE g⁻¹ DW) and “Azar Tasuj” (4.59 mg RE g⁻¹ DW). Cultivars “Sahand Ilkhchi” (4.91 mg RE g⁻¹ DW) and “Sangi Azarshahr” (5.27 mg RE g⁻¹ DW) showed intermediate levels (Fig. 4-B). The range of TFC indicates considerable variation (approximately 42% from minimum to maximum) across the tested cultivars.

### Total carotenoid content

Carotenoid accumulation also differed significantly among cultivars (F = 21.00, *p* < 0.01). “Sahand Ilkhchi” exhibited the greatest carotenoid concentration (1.81 mg β-carotene Eq. 100 g⁻¹ DW), followed closely by “Azar Tasuj” (1.65 mg β-carotene Eq. 100 g⁻¹ DW). Intermediate levels were detected in “Sangi Shahin Dej” (1.45 mg β-carotene Eq. 100 g⁻¹ DW), whereas “Sangi Azarshahr” (1.21 mg β-carotene Eq. 100 g⁻¹ DW) and “Yamatga Shabestar” (1.20 mg β-carotene Eq. 100 g⁻¹ DW) showed the lowest concentrations (Fig. 4-C).

### Total tannin content

Total tannin levels varied significantly among the almond cultivars (F = 64.15, *p* < 0.01). The highest concentration was observed in “Yamatga Shabestar” (22.54 mg TAE g⁻¹ DW), clearly distinguishing it from the other cultivars. This maximum value is more than double the minimum value observed in “Azar Tasuj”. Relatively high tannin contents were also detected in “Sahand Ilkhchi” (16.53 mg TAE g⁻¹ DW) and “Sangi Azarshahr” (13.45 mg TAE g⁻¹ DW). In contrast, “Sangi Shahin Dej” (11.48 mg TAE g⁻¹ DW) and “Azar Tasuj” (10.51 mg TAE g⁻¹ DW) exhibited comparatively lower values. This pattern reflects strong genotype-dependent tannin accumulation, with “Yamatga Shabestar” exhibiting a remarkable advantage in this trait (Fig. 4-D).

### Antioxidant activity (FRAP)

Antioxidant capacity, as measured by FRAP, revealed significant variation among cultivars (F = 1500.00, *p* < 0.01). “Yamatga Shabestar” again ranked highest, with a reducing power of 0.010 mmol Fe²⁺ g⁻¹ DW, underscoring its strong antioxidant potential. The magnitude of this difference from the mean suggests a high biological relevance for “Yamatga Shabestar”. Substantially lower values were observed in “Sahand Ilkhchi” and “Sangi Azarshahr” (0.004 mmol Fe²⁺ g⁻¹ DW), followed by “Sangi Shahin Dej” (0.003 mmol Fe²⁺ g⁻¹ DW) as shown in Fig. 4. The weakest activity was detected in “Azar Tasuj” (0.002 mmol Fe²⁺ g⁻¹ DW). The consistency of “Yamatga Shabestar” across multiple biochemical indices highlights its superior nutraceutical profile.

### Vitamin E content

The analysis of variance (ANOVA) revealed highly significant differences (F = 43.03, *p* ≤ 0.01) in vitamin E (α-tocopherol) content among the five studied almond cultivars. The highest concentration of vitamin E was recorded in the “Yamatga Shabestar” cultivar (35.34 mg 100 g⁻¹ DW), followed by “Azar Tasuj” (31.52 mg 100 g⁻¹ DW), “Sangi Shahin Dej” (28.70 mg 100 g⁻¹ DW), and “Sahand Ilkhchi” (27.34 mg 100 g⁻¹ DW). The lowest content was observed in the “Sangi Azarshahr” cultivar (18.42 mg 100 g⁻¹ DW) (Fig. 4-F).

### Determination of B-group vitamins

**Fig. 5:**
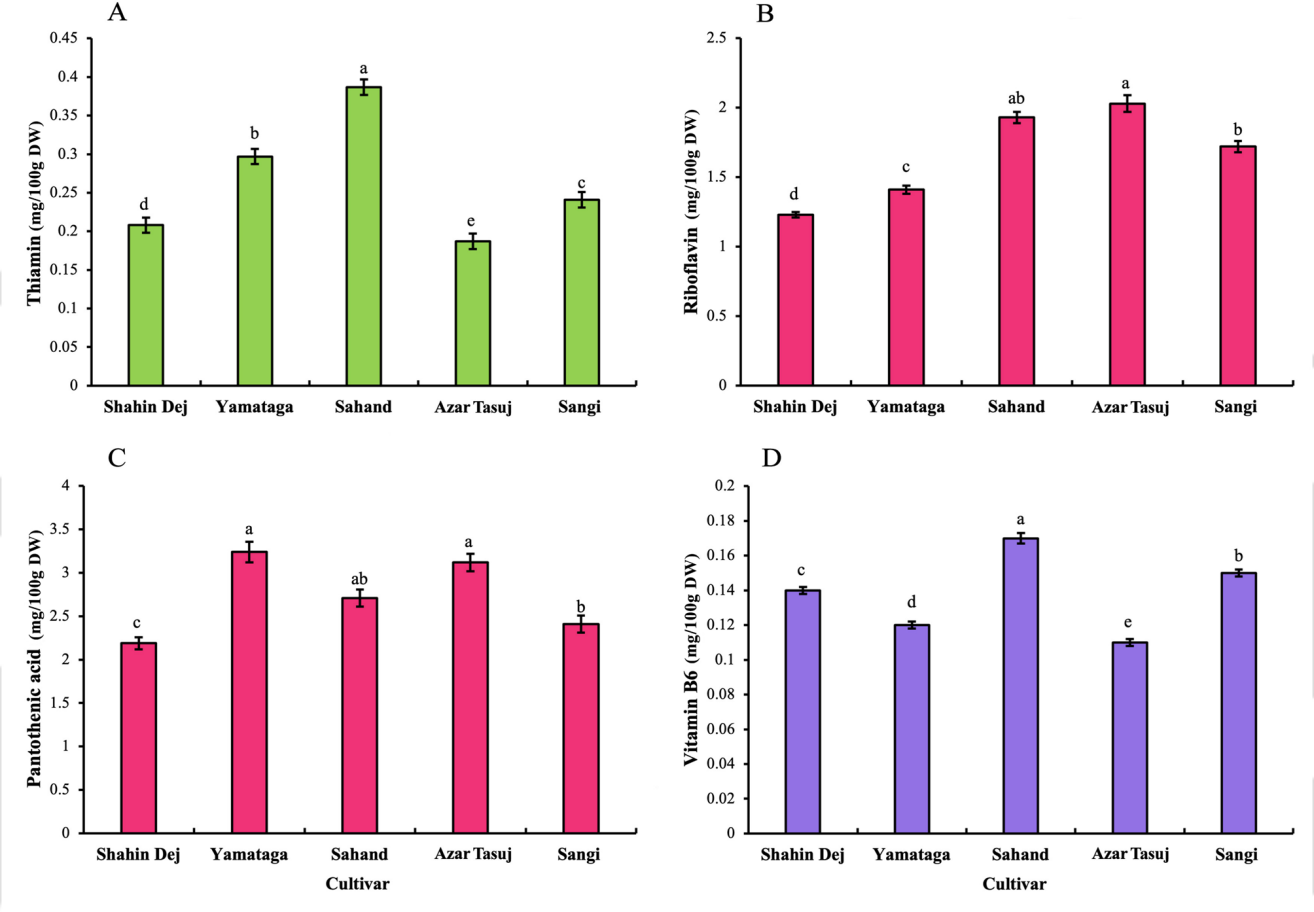
B-vitamin content of five Iranian almond cultivars. Concentrations of Thiamin (Vitamin B1, **A**), Riboflavin (Vitamin B2, **B**), Pantothenic acid (Vitamin B5, **C**), and Vitamin B6 (D) are expressed in mg/100 g DW. Error bars represent mean ± SD (*n* = 25), and different lowercase letters (a–e) indicate statistically significant differences among cultivars (*p* < 0.05, Duncan’s multiple range test).

The analysis of variance revealed a highly significant difference (F = 10.00, *p* ≤ 0.01) in thiamine (vitamin B1) content among the almond cultivars. Such variation can be attributed to both genetic background and environmental conditions influencing vitamin biosynthesis and accumulation. The highest thiamine concentration was recorded in the “Sahand Ilkhchi” cultivar (0.387 mg 100 g⁻¹ DW), followed by “Yamatga Shabestar” (0.297 mg 100 g⁻¹ DW), “Sangi Azarshahr” (0.241 mg 100 g⁻¹ DW), and “Sangi Shahin Dej” (0.208 mg 100 g⁻¹ DW). The lowest level was observed in “Azar Tasuj” (0.187 mg 100 g⁻¹ DW) (Fig. 5-A). Similarly, riboflavin (vitamin B2) content differed significantly among cultivars (F = 11.33, *p* ≤ 0.01). “Azar Tasuj” exhibited the highest concentration (2.03 mg 100 g⁻¹ DW), followed by “Sahand Ilkhchi” (1.93 mg 100 g⁻¹ DW), “Sangi Azarshahr” (1.72 mg 100 g⁻¹ DW), “Yamatga Shabestar” (1.41 mg 100 g⁻¹ DW), and “Sangi Shahin Dej” (1.23 mg 100 g⁻¹ DW) (Fig. 5-B). Significant variation (F = 12.00, *p* ≤ 0.01) was also found in pantothenic acid (vitamin B5). The maximum content was detected in “Yamatga Shabestar” (3.24 mg 100 g⁻¹ DW), while “Azar Tasuj” (3.12 mg 100 g⁻¹ DW), “Sahand Ilkhchi” (2.71 mg 100 g⁻¹ DW), “Sangi Azarshahr” (2.41 mg 100 g⁻¹ DW), and “Sangi Shahin Dej” (2.19 mg 100 g⁻¹ DW) ranked next, as shown in Fig. 5-C. For pyridoxine (vitamin B6), a statistically significant difference was observed across the cultivars (F = 10.00, *p* ≤ 0.01). The highest content was recorded in “Sahand Ilkhchi” (0.17 mg 100 g⁻¹ DW), followed by “Sangi Azarshahr” (0.15 mg 100 g⁻¹ DW), “Sangi Shahin Dej” (0.14 mg 100 g⁻¹ DW), “Yamatga Shabestar” (0.12 mg 100 g⁻¹ DW), and “Azar Tasuj” (0.11 mg 100 g⁻¹ DW) (Fig. 5-D). This significant trait-specific variation in B-vitamins demonstrates that different cultivars are superior for different nutritional compounds.

### Phenolic profile and quantification by HPLC

**Fig. 6:**
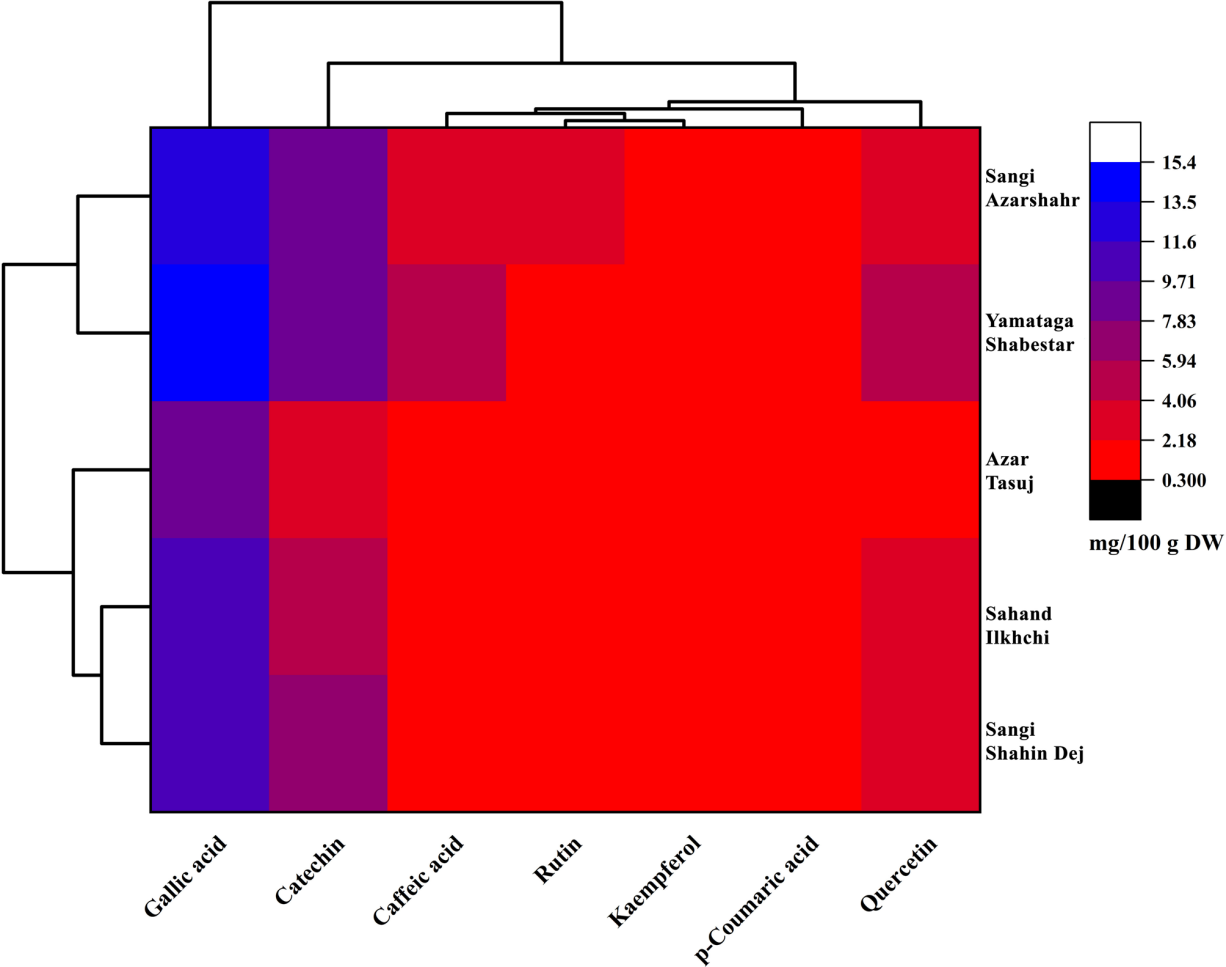
almond cultivars. Concentrations of Gallic acid, Catechin, Caffeic acid, Rutin, Kaempferol, p-Coumaric acid, and Quercetin were quantified using HPLC-DAD and are expressed in mg/100 g DW. The color intensity on the heatmap reflects compound concentration, while dendrograms depict hierarchical clustering of cultivars (left) and compounds (top) based on their profiles. This visualization highlights cultivar-specific phytochemical fingerprints and the metabolic diversity among the studied almonds.

To evaluate the phenolic composition of almond cultivars, seven phenolic compounds—gallic acid, catechin, caffeic acid, rutin, kaempferol, *p*-coumaric acid, and quercetin—were quantified using high-performance liquid chromatography (HPLC). The results are visualized as a heat map (Fig. 6), where color intensity reflects the concentration of each compound (mg 100 g⁻¹ DW) across the cultivars. The highest gallic acid content was recorded in “Yamatga Shabestar” (13.50 mg 100 g⁻¹ DW), representing the maximum accumulation among the studied cultivars, whereas the lowest level was observed in “Azar Tasuj”. This substantial difference suggests a strong genetic control over this specific metabolite. Catechin concentration was greatest in “Sangi Azarshahr”, while it remained particularly low in “Sangi Shahin Dej” and “Azar Tasuj”. In contrast, caffeic acid showed its highest accumulation in “Yamatga Shabestar”, whereas the other cultivars contained relatively lower amounts. Rutin, kaempferol, *p*-coumaric acid, and quercetin were generally present at lower concentrations across most cultivars. However, quercetin peaked in “Yamatga Shabestar” (4.06 mg 100 g⁻¹ DW), highlighting its distinct phenolic profile. Cluster analysis based on phenolic composition revealed a clear differentiation among the studied cultivars. Notably, “Yamatga Shabestar” was distinctly separated from the other cultivars, which appears to be mainly related to its higher levels of gallic acid and quercetin, together with its overall richer phenolic profile. In contrast, the cultivars grouped within the low-phenolic cluster showed generally lower concentrations of these compounds, while “Sangi Azarshahr” was mainly characterized by its relatively higher catechin content.

### Multivariate analysis (cluster and PCA)

**Fig. 7:**
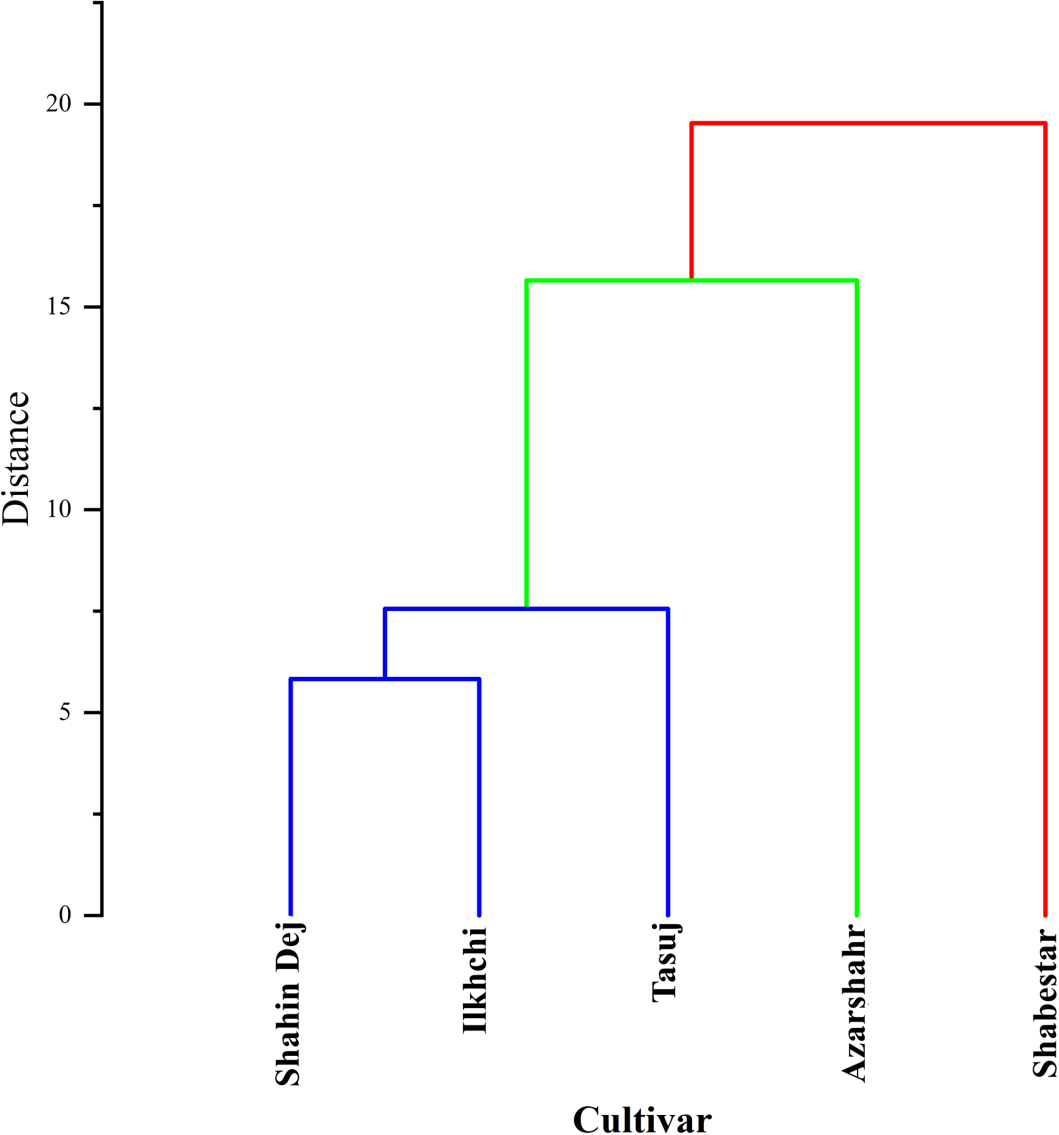
Dendrogram from hierarchical cluster analysis (HCA) of five Iranian almond cultivars. The dendrogram depicts relationships and genetic distances based on combined physical and biochemical traits. Shahin Dej and Ilkhchi form a closely related cluster, whereas Azarshahr and Shabestar cluster together but are more distantly related to the other cultivars, highlighting the genetic and metabolic diversity among the studied almonds.

Cluster analysis based on morphological and biochemical traits grouped the five cultivars into three main clusters (Fig. 7). “Sangi Shahin Dej”, “Sahand Ilkhchi”, and “Azar Tasuj” formed one group characterized mainly by similarities in fruit and kernel dimensions. “Sangi Azarshahr” was positioned separately due to its intermediate biochemical profile. In contrast, “Yamatga Shabestar” formed a distinct cluster, reflecting its superior accumulation of phenolic compounds, vitamins, and antioxidant-related traits.

**Fig. 8:**
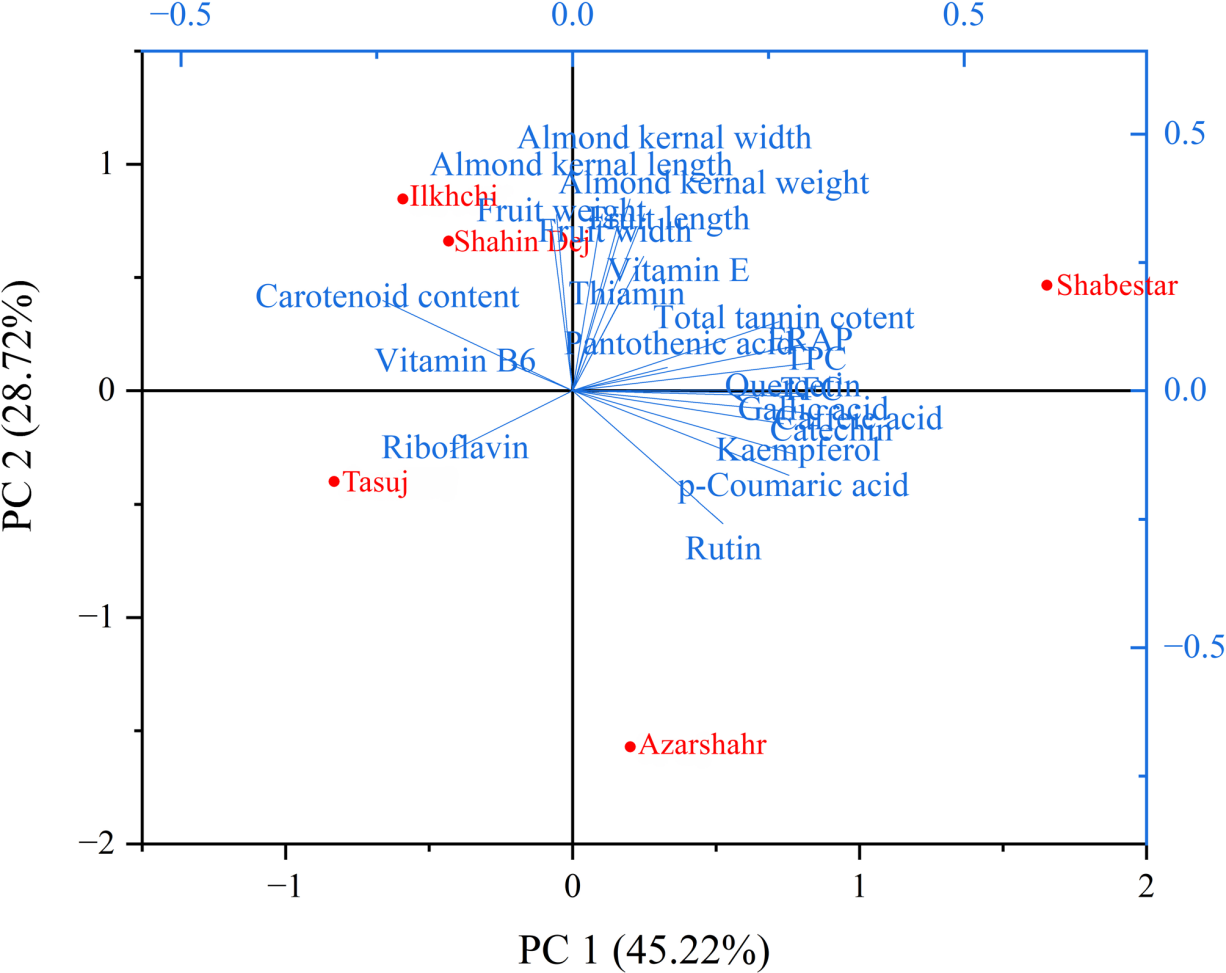
Principal component analysis (PCA) biplot of physical and biochemical traits in five Iranian almond cultivars. Cultivars are indicated by red circles, and measured traits are represented by blue vectors. The first two principal components, PC1 (45.22% variance) and PC2 (28.72% variance), together explain a substantial proportion of the total variability. The proximity of a cultivar to a vector indicates a strong association with that trait, while the angles between vectors reflect correlations among traits (small angles indicate positive correlation, large angles negative correlation, and 90° indicates no correlation).

Principal component analysis (PCA) supported this classification (Fig. 8). The first two components explained 73.94% of the total variance (PC1 = 45.22%, PC2 = 28.72%). The distribution of cultivars along PC1 largely reflected variation in phenolic and antioxidant-related variables. “Yamatga Shabestar” was positioned on the positive side of PC1, in the general direction of total phenolics (TPC), FRAP, total tannins, gallic acid, quercetin, and caffeic acid, suggesting its contribution to this biochemical gradient. “Sangi Azarshahr” was separated mainly along the negative axis of PC2, indicating differentiation from the other cultivars along this component rather than a specific association with a single metabolite. In contrast, “Azar Tasuj” appeared closer to the riboflavin loading vector and showed partial alignment with carotenoid content and vitamin B6. The remaining cultivars were positioned nearer to morphological variables, suggesting a relatively stronger contribution of physical traits to their multivariate placement. Overall, multivariate analysis confirmed the metabolic distinctiveness of “Yamatga Shabestar” and highlighted clear differentiation among cultivars based on combined morphological and biochemical characteristics.

### Correlation analysis among morphological, biochemical, and phenolic traits in almond cultivars

**Fig. 9:**
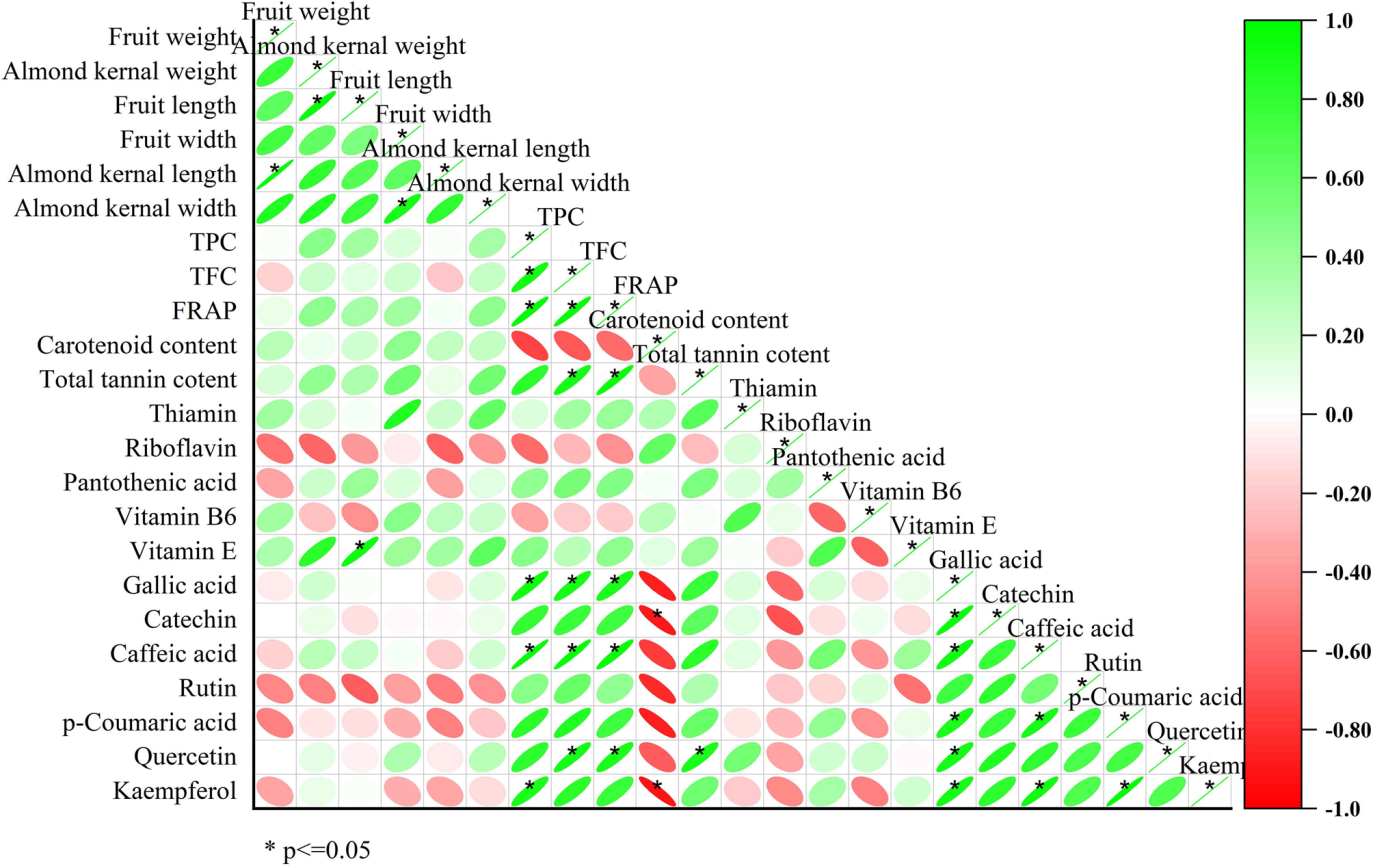
Correlation matrix of physical and biochemical traits in Iranian almond cultivars. The matrix is visualized as a heatmap of ellipses, where the color and shape represent the strength and direction of the correlation. Green ellipses indicate positive correlations, red ellipses indicate negative correlations, and the elongation of each ellipse reflects the correlation magnitude (a perfectly flat line indicates r = ± 1, a circle indicates *r* = 0). Statistically significant correlations (*p* < 0.05) are marked with a star (*).

The correlation heatmap (Fig. 9) revealed significant associations among morphological, biochemical, and phenolic variables. Strong positive correlations were observed among fruit weight, kernel weight, and fruit dimensions, indicating coordinated morphological development across cultivars. Among biochemical traits, total phenolic content (TPC), total flavonoid content (TFC), total tannins, and FRAP were positively correlated, confirming the major contribution of phenolic compounds to antioxidant capacity. TPC showed a particularly strong association with FRAP, supporting its role as a primary determinant of reducing power. Carotenoids exhibited negative correlations with several phenolic traits, suggesting differential metabolic allocation between phenylpropanoid- and terpenoid-derived compounds. B-group vitamins showed limited or variable correlations among themselves, whereas vitamin B6 exhibited a significant positive association with carotenoid content. Vitamin E showed a mild positive tendency with FRAP and gallic acid; however, these associations were not statistically significant. Within the phenolic profile, gallic acid, catechin, and caffeic acid were strongly intercorrelated, reflecting shared biosynthetic origin, while quercetin and kaempferol were positively associated within the flavonol branch. Overall, cultivars characterized by high TPC and FRAP (notably “Yamatga Shabestar”) tended to accumulate higher levels of key phenolic compounds, whereas carotenoid-rich cultivars followed a distinct metabolic pattern.

## Discussion

The findings of the present study demonstrate significant variability among almond cultivars in terms of morphological, biochemical, and antioxidant traits, emphasizing the strong influence of both genetic background and potential environmental adaptation mechanisms. Such cultivar-dependent differences are consistent with previous studies in *Prunus* species, where both primary and secondary metabolites exhibited high inter-cultivar variability^32,33^. The observed patterns provide preliminary insights into the nutritional and functional profiles of the evaluated cultivars and may inform future breeding-oriented investigations, although confirmation under multi-year and multi-environment conditions is required.

Environmental heterogeneity among sampling sites, including differences in altitude, long-term mean temperature, soil characteristics, and average rainfall (Table 1), represents background agro-ecological variation that may contribute to phytochemical diversity. However, the present study was not designed to specifically evaluate environmental stress effects, and no unusual climatic conditions were recorded for the sampling year. Therefore, environmental influences should be interpreted cautiously and within the context of genotype–site association^34^. It should be noted that in the present study, each almond cultivar was sampled from a single representative cultivation region. Consequently, the effects of genotype and environment cannot be fully disentangled, and the observed differences among cultivars reflect the combined influence of genetic background and site-specific environmental conditions. This limitation should be considered when interpreting cultivar-dependent variation, particularly in the context of genotype × environment interactions.

Substantial variation was observed across all measured morphological traits, including fruit weight, kernel weight, fruit length, fruit width, kernel length, and kernel width. The superior fruit weight and kernel length of “Sangi Shahin Dej”, along with the high kernel weight and fruit length of “Yamatga Shabestar”, reflect differential genetic allocation between endosperm and pericarp development. These differences may be related to distinct sink–source dynamics, as well as differences in carbohydrate assimilation efficiency during nut filling^35,36^. Conversely, the consistently low values in “Azar Tasuj” suggest either a reduced assimilate partitioning efficiency or inherently smaller seed size. Such morphological variability is not only important from a commercial perspective, given that fruit and kernel size strongly affect marketability, but also indicates the potential genetic diversity that can be exploited for breeding programs aimed at both yield and quality improvement^37^.

The observed differences in total phenolic content among cultivars may reflect genotype-dependent variation in secondary metabolite regulation^38^. The higher accumulation detected in “Yamatga Shabestar” may indicate differences in phenylpropanoid-related metabolic activity; however, no molecular or enzymatic analyses were conducted to directly verify this mechanism. Therefore, this interpretation should be considered as a preliminary data survey, useful for future investigations^39^. Phenolic compounds are major contributors to the antioxidant and nutraceutical potential of almonds^40^. In the present study, total phenolic content (TPC) varied significantly among cultivars, ranging from 0.12 mg GAE g⁻¹ DW in “Azar Tasuj” to 1.07 mg GAE g⁻¹ DW in “Yamatga Shabestar”. The exceptionally high F values observed for parameters such as TPC and FRAP are attributable to pronounced between-cultivar differences combined with very low within-cultivar variance, as reflected by the low coefficients of variation (CV < 5%). This pattern indicates strong genotype-dependent divergence together with high experimental consistency rather than statistical artefacts. Comparable variability has been reported in previous investigations on almond germplasm across different regions. For example, total phenolic content (TPC) values ranging from 1.27 to 2.41 mg GAE g⁻¹ FW have been documented among major Californian cultivars, highlighting significant genotype-dependent variation^28^. In Mediterranean almonds, particularly in Avola varieties and related industrial cultivars, further variability has been observed in phenolic concentration and composition^41^. In addition, almond skins have been reported to account for approximately 60–80% of the total phenolics of the whole fruit, confirming the strong tissue-specific distribution of these compounds^42^. The TPC values observed in the present work therefore fall within the spectrum reported in the literature, and the concentration measured in “Yamatga Shabestar” appears to be positioned toward the upper range reported for comparable traditional landraces. Differences among studies may be attributed to genetic background, agro-climatic conditions, kernel processing (with or without skin), and solvent extraction systems. The superior phenolic accumulation observed in “Yamatga Shabestar” may reflect differences in phenylpropanoid-related metabolic regulation. Although enhanced activity of phenylalanine ammonia-lyase (PAL) and other enzymes of the phenylpropanoid pathway has been associated with increased phenolic biosynthesis in *Prunus* species^43^, no direct enzymatic or molecular analyses were conducted in the present study to verify this mechanism. Flavonoids, representing a major subclass of almond phenolics, also exhibited pronounced cultivar-dependent variation. Comparable patterns have been reported previously. For example, almonds with intact skins were shown to contain 2.39 mg GAE g⁻¹ FW, whereas removal of the skin reduced this value to 0.47 mg GAE g⁻¹ FW, underscoring the dominant contribution of flavonoid-rich seed coats to total phenolics^44^. Similarly, Mandalari et al.^45^ demonstrated that natural almond skins possess significantly higher phenolic concentrations than blanched skins due to processing-induced leaching into blanch water, confirming that both genotype and technological treatments influence flavonoid recovery. The elevated TFC observed in “Yamatga Shabestar” is consistent with previously reported quantitative ranges and aligns with its higher FRAP values^2^. Given that flavonoids are well recognized for their reducing power and radical-scavenging capacity in almond-derived matrices^46^, the association between TFC and antioxidant performance observed here is biologically consistent with established antioxidant mechanisms. Overall, the magnitude of inter-cultivar variability observed among the evaluated Iranian genotypes appears comparable to that reported internationally. The contrasting carotenoid distribution may further suggest potential genotype × environment (G×E) influences on metabolic allocation between phenolic and terpenoid pathways.

Carotenoid accumulation also differed significantly among cultivars in the present study. The recorded concentrations (1.20–1.81 mg β-carotene equivalents 100 g⁻¹ DW) indicate measurable inter-cultivar variability, with “Sahand Ilkhchi” exhibiting the highest values and “Yamatga Shabestar” the lowest. Comparable variability has been reported in previous investigations. Almond kernels are generally characterized by relatively low but nutritionally relevant carotenoid concentrations, typically ranging between approximately 0.5 and 3.0 mg 100 g⁻¹ DW depending on genotype, maturity stage, and analytical method. These values are broadly consistent with previous reports; for instance, carotenoid content in almond oil has been reported to be as low as 5–8 mg kg⁻¹^47,48^. Differences between studies may be attributed to variations in sample type (kernel vs. oil), extraction methods, and expression units, which can influence the reported concentrations. Therefore, the carotenoid levels observed in the present study fall within the expected range reported in the literature. In Mediterranean almond cultivars, variation in carotenoid content has also been associated with cultivar-specific traits and environmental conditions, including light intensity and temperature^49^. Such factors are known to modulate carotenoid biosynthesis and pigment stability in nut crops. Because each genotype in the present study was sampled from a distinct geographical location, the observed differences likely reflect the combined influence of genetic background and local agro-climatic conditions rather than exclusively intrinsic metabolic capacity. Functionally, carotenoids act as lipid-soluble antioxidants and precursors of vitamin A, contributing to both photoprotection and nutritional value^50^. Because carotenoids and phenolics originate from distinct biosynthetic pathways, the contrasting accumulation patterns observed among cultivars likely reflect differential metabolic regulation. Overall, the carotenoid concentrations reported here fall within the spectrum described for almond kernels in previous international studies, suggesting that the magnitude of inter-cultivar variability observed among these Iranian landraces is consistent with globally documented patterns.

The ferric reducing antioxidant power (FRAP) assay revealed significant differences in antioxidant capacity among the evaluated cultivars, with “Yamatga Shabestar” displaying the highest reducing power. This observation is consistent with its elevated total phenolic (TPC) and total flavonoid (TFC) contents, supporting the strong association between phenolic accumulation and antioxidant potential^51^. “Sahand Ilkhchi” and “Sangi Azarshahr” exhibited intermediate antioxidant activity, whereas “Azar Tasuj” recorded the lowest FRAP value. These findings align with previous reports indicating that phenolic compounds, particularly flavonoids and hydroxycinnamic acids, contribute substantially to reducing capacity compared with other phytochemical groups such as carotenoids^52^. Accordingly, the enhanced FRAP value observed in “Yamatga Shabestar” is likely attributable to its higher phenolic and flavonoid concentrations.

Although the FRAP assay provides a reliable estimate of total reducing capacity, it primarily reflects electron-donating ability and does not encompass all antioxidant mechanisms, such as radical scavenging or hydrogen atom transfer. Therefore, the reported values should be interpreted as an index of reducing power rather than a comprehensive assessment of antioxidant activity. The incorporation of complementary assays (e.g., DPPH or ABTS) in future investigations would enable a more complete characterization of antioxidant properties.

Tannins, another group of phenolic compounds with notable bioactive properties, also varied significantly among cultivars. “Yamatga Shabestar” exhibited the highest tannin levels, followed by “Sahand Ilkhchi”, while “Azar Tasuj” had the lowest. This trend parallels TPC to some extent, reinforcing the idea that “Yamatga Shabestar” is metabolically geared toward higher phenolic accumulation. Tannins are known to contribute to both antioxidant and antimicrobial properties, and their high levels in certain cultivars may enhance the potential nutraceutical value of these almonds^53,54^. It is important to clarify that total phenolic content (TPC), total flavonoids (TFC), and total tannins were quantified using independent colorimetric assays based on different chemical principles and calibration standards (GAE, RE, and TAE, respectively). These methods do not measure identical chemical entities and are not directly additive. The Folin–Ciocalteu assay estimates overall reducing capacity rather than exclusively phenolic compounds, while tannin quantification at 280 nm may overestimate aromatic constituents due to its lower specificity. Therefore, apparent discrepancies in magnitude between TPC and tannin or flavonoid values should not be interpreted as literal quantitative inconsistencies, but rather as methodological differences inherent to spectrophotometric assays.

Significant cultivar-dependent variation was observed for B-group vitamins and α-tocopherol. “Yamatga Shabestar” showed the highest vitamin E and pantothenic acid levels, whereas other cultivars were superior for specific B vitamins. These differences highlight genotype-driven variability in micronutrient accumulation and may be relevant for nutritional selection.

HPLC profiling confirmed the presence of seven key phenolic compounds, gallic acid, catechin, caffeic acid, rutin, kaempferol, p-coumaric acid, and quercetin, with distinct cultivar-dependent distribution patterns. “Yamatga Shabestar” exhibited the highest contents of gallic acid and caffeic acid, while catechin was most abundant in “Sangi Azarshahr”, and quercetin accumulation was also prominent in “Yamatga Shabestar”. This suggests that although “Yamatga Shabestar” almond exhibits superior overall phenolic richness, specific phenolic compounds are preferentially accumulated in other cultivars, indicating differential regulation within the shikimate and phenylpropanoid biosynthetic pathways^55^. The quantitative values of B-group vitamins and vitamin E obtained in the present study were further evaluated in comparison with official food composition databases. According to the USDA FoodData Central database^56^, raw almonds contain approximately 0.16 mg 100 g⁻¹ of thiamine (vitamin B1) and 0.10 mg 100 g⁻¹ of vitamin B6 on an edible portion basis. In the present study, thiamine content ranged from 0.19 to 0.39 mg 100 g⁻¹ DW, while vitamin B6 ranged from 0.11 to 0.17 mg 100 g⁻¹ DW across cultivars. These values are generally higher than the USDA reference data, which may be attributed to genotype-specific variation as well as differences in expression basis (dry weight versus fresh edible portion). Similarly, the Italian CREA Food Composition Database^57^ reports approximately 26 mg 100 g⁻¹ of vitamin E (α-tocopherol) in almonds. In contrast, the cultivars examined in the present work exhibited α-tocopherol concentrations ranging from 18.4 to 35.3 mg 100 g⁻¹ DW, with “Yamatga Shabestar” exceeding the database value. Such variation highlights the significant impact of cultivar selection and local agro-climatic conditions on micronutrient accumulation.

It should be noted that official database values are typically expressed on a fresh edible portion basis, whereas the present results are reported on a dry weight basis. This methodological difference, together with genotype-dependent metabolic variability, may partially explain the observed quantitative discrepancies. Overall, the vitamin concentrations measured in this study fall within, and in some cases exceed, internationally reported ranges, supporting the nutritional relevance of the evaluated Iranian almond landraces.

The higher levels of quercetin, catechin, and gallic acid in “Yamatga Shabestar” may partially explain its superior FRAP values. These compounds are widely recognized contributors to antioxidant capacity in almond kernels. However, given the complexity of the almond matrix, additional unquantified compounds may also contribute to the observed activity^58^. The elevated levels of these compounds in “Yamatga Shabestar” likely underpin its superior antioxidant capacity measured by FRAP, highlighting the strong correlation between specific phenolic composition and overall radical-scavenging potential. Moreover, the cultivar-dependent differences in metabolite accumulation may also reflect genotype ×environment interactions, as environmental factors such as altitude, temperature, and soil properties can influence enzyme activity in key biosynthetic pathways, ultimately shaping the phenolic profile of each cultivar^57^. Taken together, these results emphasize that both genetic background and adaptive metabolic regulation contribute to the observed diversity in phenolic content and antioxidant activity among almond cultivars, offering insights into the selection of nutritionally and functionally superior genotypes for breeding and functional food applications. Although ‘Yamatga Shabestar’ demonstrated superior biochemical traits, its practical deployment for large-scale cultivation or commercial processing requires further evaluation of agronomic yield, pest resistance, and post-harvest stability. Future breeding efforts should balance these biochemical advantages with field performance and consumer acceptability. Multivariate analyses confirmed the distinct biochemical profile of “Yamatga Shabestar”, which was consistently associated with phenolic and antioxidant-related traits. This statistical separation supports its metabolic divergence relative to the other cultivars. A critical discussion of assay limitations is necessary to contextualize our findings. Assays used for total quantification, such as TPC and FRAP, are non-selective; they measure a broad range of compounds (including reducing sugars and amino acids) that may contribute to the final value, potentially leading to overestimation of the true phenolic contribution^59^. Therefore, the high TPC/FRAP values should be interpreted cautiously as an overall indicator of reducing capacity, and not solely attributed to phenolics. Furthermore, our HPLC profiling was limited to seven known compounds; while these are important markers, it is highly likely that other phenolics present in the almond matrix (such as complex proanthocyanidins) were not quantified, meaning the full phytochemical profile remains uncharacterized.

Finally, while the nutraceutical profile of ‘Yamatga Shabestar’ is highly promising for functional food development, its commercial implementation is subject to practical constraints. We acknowledge that this study does not provide data on key agronomic parameters such as kernel yield per tree/hectare, resistance to major pests (e.g., Fusicoccum), disease susceptibility, or specific post-harvest stability. Without detailed agronomic and economic assessment, its utility remains primarily a valuable genetic resource for breeding rather than an immediately marketable commercial product. Because each cultivar was evaluated in a single location and single growing season, the present results should be interpreted as exploratory. Future studies incorporating multi-location and multi-year trials are necessary to improve the robustness of genotype characterization and to disentangle genotype × environment interactions. In addition, although five biological replicates (trees) were included per cultivar, the total number of fruits analyzed per genotype was relatively limited, which may restrict the full representation of intra-cultivar variability. Expanding the sampling size in future investigations would further enhance statistical robustness and strengthen the reliability of cultivar-specific conclusions.

## Conclusion

The present study provides a comprehensive comparative evaluation of the phytochemical composition and antioxidant properties of five Iranian almond cultivars. Significant genotypic variation was detected, with “Yamatga Shabestar” distinguished by its exceptionally high levels of α-tocopherol (35.34 mg/100 g DW), total phenolics, flavonoids, and tannins, consistently conferring the strongest antioxidant activity. Positive correlations between these bioactive compounds and antioxidant capacity highlight their synergistic contribution to almond kernel health benefits. These results emphasize the critical role of genetic background in shaping the nutraceutical value of almonds and represent the first detailed comparison of Iranian almond germplasm in this regard. The findings position “Yamatga Shabestar” as a superior genetic resource for immediate use in breeding programs aimed at enhancing human health benefits. However, the observed superiority of “Yamatga Shabestar” is context-dependent, and the generalizability of these findings is limited by the single-site study design. For broader commercial application, the superior biochemical profile must be confirmed through multi-environmental trials and balanced against essential agronomic factors such as yield, disease resistance, and consumer acceptance.

The detailed comparative data generated here provides a strong mechanistic foundation for future investigation. Integrating advanced genomic and metabolomic tools will be essential to precisely clarify the specific regulatory mechanisms underlying the observed metabolic divergence and to guide the development of optimized cultivation strategies for maximizing almond nutraceutical potential.

## Supplementary Information

Below is the link to the electronic supplementary material.

Supplementary Material 1

## Data Availability

All data generated or analyzed during this study are included in this article. Further enquiries can be directed to the corresponding author.
