# Supplementary material for: Phytochemical profile and antioxidant activity of almond (Prunus amygdalus Batsch.) cultivars from northwestern Iran
Source: Sci Rep. 2026 Apr 25;16:19134. doi: 10.1038/s41598-026-50377-6 (PMC13280246; doi:10.1038/s41598-026-50377-6)
Supplement: Supplementary file 1 — Supplementary Material 1 [file 41598_2026_50377_MOESM1_ESM.docx]

Table S1. Calibration parameters of phenolic standards used for HPLC quantification

| **Compound** | **Calibration range (ppm)** | **Calibration equation** | **R²** |
| --- | --- | --- | --- |
| Chlorogenic acid | 5–250 | Y = 52,375X + 35,555 | 0.9981 |
| Gallic acid | 5–250 | Y = 123,521X − 716,832 | 0.9971 |
| Rutin | 5–250 | Y = 39,917X − 162,112 | 0.9989 |
| Caffeic acid | 5–250 | Y = 200,397X − 1,000,006 | 0.9978 |
| 3,4-Dihydroxybenzoic acid | 5–250 | Y = 26,816X − 511,178 | 0.9875 |
| Ferulic acid | 5–250 | Y = 209,112X − 875,151 | 0.9844 |
| Rosmarinic acid | 5–250 | Y = 257,194X − 234,172 | 0.9873 |
| Quercetin | 5–250 | Y = 70,624X − 241,366 | 0.9997 |
| p-Coumaric acid | 5–250 | Y = 259,208X − 712,294 | 0.9953 |
| Vanillic acid | 5–250 | Y = 118,522X − 263,440 | 0.9915 |
| Salicylic acid | 5–250 | Y = 16,577X − 18,949 | 0.9995 |
| Benzoic acid | 5–250 | Y = 112,833X − 558,841 | 0.9889 |
| Syringic acid | 5–250 | Y = 145,598X − 653,099 | 0.9941 |
| Kaempferol | 5–250 | Y = 8,966X − 48,817 | 0.9981 |

Calibration curves were constructed using external standards over the concentration range of 5–250 ppm. Y represents peak area and X represents standard concentration.
